# Brain Death and Management of Potential Organ Donor: An Indian Perspective

**DOI:** 10.5005/jp-journals-10071-23194

**Published:** 2019-06

**Authors:** Kapil Zirpe, Sushma Gurav

**Affiliations:** 1,2 Department of Neurotrauma, Ruby Hall Clinic, Pune, Maharashtra, India

## Abstract

**How to cite this article:** Zirpe K, Gurav S. Brain Death and Management of Potential Organ Donor: An Indian Perspective. Indian J Crit Care Med 2019;23(Suppl 2):S151–S156.

## INTRODUCTION

Organ donation is the most rewarding medical care which has saved many lives. The organ donation rate in India have increased from a dismal 0.05 per million population to 0.8 per million population in a span of few years.^1^ Organ donation rates in India are minuscule compared to Croatia's 36.5, Spain's 35.3, and America's 26 per million, respectively. The vast difference between the demand for organs and their poor supply is the main issue of concern.^2^

Over 147,913 fatalities were attributed to road traffic accidents in India, in the year 2017.^3,4^

In nearly 40–50% of road accident fatalities, the cause of death was head injury.

If 5–10% of all brain-dead patients are considered for organ harvesting, there would be no requirement for a living person to donate organs.^2^

In 1994, brainstem death was legalized in India. The Transplantation of Human Organs (THO) Act of 1994 and the subsequent amendments in 2011 and 2014 form the legislative foundation for brain death declaration and organ donation.^5-7^ The criteria for brainstem death declaration in our country is based on United Kingdom guidelines.^5-7^

Because all the potential donor enter ICU at some point of time, intensivist have important role in giving care to potential organ donor^8^ (Table 1).

### Who is Potential Brain-dead Donor (PBDD)?

A potential organ donor is defined by the presence of either brainstem death or a catastrophic and irreversible brain injury that leads to fulfilling the brainstem death criteria.^9^

### What is Brainstem Death?

“Brainstem death” means the stage at which all brain functions are permanently and irreversibly ceased. However, the cause of irreversible coma has to be established, preconditions should be met, and confounding factors are to be ruled out.

### Care of Potential Organ Donor

Organ donation system requires early identification of PBDD and early appropriate evaluation and conversion of PBDD to actual donors. Up to 20% of organs and a large number of PBDDs are lost because the clinical management is challenging. This can be overcome with the use of bedside checklists to achieve cardiovascular, respiratory and endocrine-metabolic targeted physiology. Adequate time should be given for organ optimization and to come out of the autonomic storm injury. A median time of around 48 hours from autonomic storm to cardiac function recovery has been proven by serial echocardiographic. Ignacio Martin-Loeches et al, has recommended that organ procurement should be done within 30 hours after brainstem death. This prevents loss of potential donors, organs secondary to cardiac arrest.^10^

**Table 1:** Role of Intensivist in care of potential brain-dead patient

Maintaining hemodynamic stability
Diagnosing neurological death
Preparing the family for devastating news
Counselling family: end of life (EOL)
Implementing policies and protocols for option of organ donation
Communication with transplant coordinator, organ procurement team, family members

A protocolized and multisystem ABC approach for organ donation is being recommended^10^ (Flowchart 1 and Table 2).

Our aim should not be only to increase the number of donated organs but also to increase their quality and to reduce cardiac arrests in PBDD. This will also preserve long-term graft function.^5,10^

A simple method to maintain potential donor is “rule of 100”^11^ (Table 3).

## PATHOPHYSIOLOGY

Severe trauma or stroke causes damage to neuronal tissue. This may to lead to edema and increase in intracranial pressure (ICP) and thereby compromising cerebral perfusion. A vicious cycle is intiated, in which decreasing cerebral perfusion and increasing ICP interact with each other until no further blood flows in the cranial cavity and transtentorial or transforaminal herniation ensues. This causes damage to brainstem.

Transtentorial herniation causes damage to brainstem in rostral to caudal path, i.e., first pons is damaged and then medulla. Initial response is hypertension and bradycardia. This **phase** of brain death process called as Cushing's reflex. As the brainstem becomes ischemic, the vagal cardiomotor nucleus becomes ischemic causing unopposed sympathetic stimulation, and thereby leading to tachycardia, hypertension, and high blood levels of catecholamines (i.e., autonomic storm or catecholamine surge). This sympathetic surge, is an adaptive response to maintain cerebral perfusion pressure. But this surge causes ischemic insult to other organs – kidney, liver and heart.

**Flowchart 1:**
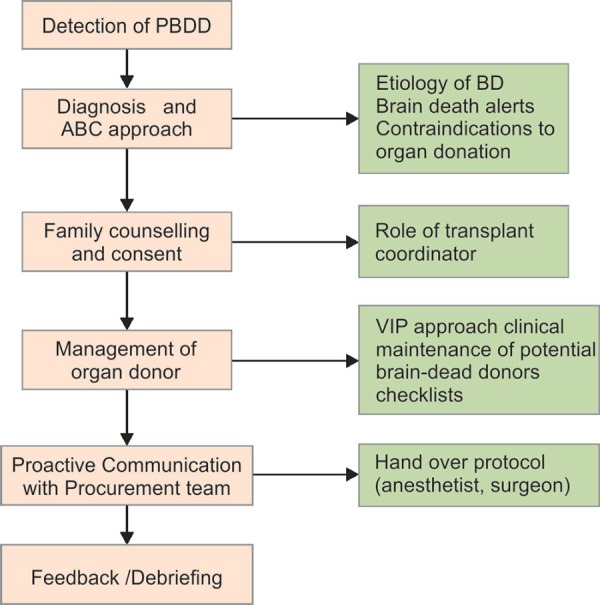
ABC approach and care of organ donor in brain death

This phase is followed by a profound reduction in sympathetic outflow, with loss of autonomic tone resulting in vasodilatation and hypotension. Concurrent ischemic damage to the hypothalamus and pituitary results in temperature and endocrine dysfunction. Thus, there is an interruption in hypothalamic-pituitary-adrenocortical regulation. During this period, circulation must be supported, respiration is artificially maintained and normal physiological sequel of brain death should be anticipated and corrected.^5,12^

## LABORATORY WORKUP: PATIENT HEIGHT WEIGHT AND CHEST CIRCUMFERENCE NEED TO BE DOCUMENTED^5^

Parameters such as blood grouping and typing, complete hemogram, blood glucose, urine analysis, blood urea nitrogen, serum creatinine, serum electrolytes, liver function tests, coagulation profile should be assessed. Serological testing for HIV, HSV antibody, HBV surface antigen, core antibody, surface antibody, HCV antibody, and IgM and IgG for cytomegalovirus are necessary.

Arterial blood gas, lactate, electrolytes, and blood sugar levels need to be monitored every 2–4 hourly.

Infection screening: Blood cultures endotracheal tube secretions and urine cultures may be required, if there is evidence of infection or if the patient is hospitalized for more than 72 hours.

Additional tests may be required for multiorgan donors, for example, echocardiography for heart, and bronchoscopy for lung transplantation.

**Table 2:** ABC care for organ donor

A:	Etiology of brainstem death or irreversible coma
B:	Brain death alert signs
C:	Contraindication to organ donation

**Table 3:** ‘Rule of 100’

Systolic arterial pressure >100 mm Hg, Urine output >100 mL/hour PaO_2_ >100 mm Hg Hemoglobin concentration >100 g/L (10 gm/dL) Blood sugar 100 mg/dL

## PHYSIOLOGICAL AND METABOLIC CHANGES IN BRAIN DEATH PATIENTS

### Cardiovascular System

Hemodynamic instability and cardiac dysfunctions are always encountered in patients after brain death. The“sympathetic storm”results in hypertension, tachycardia, and arrhythmias.

Though usually of short duration, it may lead to cardiac dysfunction, cardiac ischemia, myocardial and conduction system necrosis. Further, spinal cord ischemia is followed by deactivation of sympathetic storm and loss of cardiac stimulation. This leads to vasodilatation and cardiac dysfunction, clinically presenting as hemodynamic instability in potential donor. Use of diuretics (mannitol), osmotic diuresis due to hyperglycemia, diabetes insipidus (DI), hypothermic “cold” diuresis, inadequate fluid resuscitation, decreased oncotic pressure after crystalloid resuscitation, ongoing blood loss, rewarming of patient, relative adrenal insufficiency as a result of trauma, and critical illness can all aggravate hypotension and impair organ perfusion.^5^

Thus circulatory shock is a consequence of the impairment of at least one of the three determinants of cardiac output (preload, contractility and afterload).

So modified version of VIP approach can be used for medical management of organ donor care adjusting mechanical ventilation, fluid and drug Infusions, and maintaining heart function^10^ (Flowchart 2).

#### Hemodynamic Management (Flowchart 2)

Goals for management of hemodynamic status of the donorare as follows:

- To maintain normovolemia/euvolemia
- Optimize cardiac output so as to maintain perfusion pressure of all organs with minimal vasoactive support

#### Hypertension

It occurs due to the transient nature of autonomic storm. This causes hypertension, tachycardia and thereby increase in myocardial oxygen consumption. Antihypertensives are usually not required. If needed, short-acting antihypertensives such as esmolol, sodium nitroprusside, hydralazine, labetalol, or nitroglycerine should be used.^5^

#### Hypotension

The suitable organs for harvesting and transplantation should be identified in advance to plan subsequent focused medical management including fluid replacement. The decision for selection of particular fluid depends on serum electrolytes, glycemic control, hemodynamic status of the patient, estimated volume deficiency, and presence of polyuria from DI. Three strategies are suggested:^5^

**Flowchart 2:**
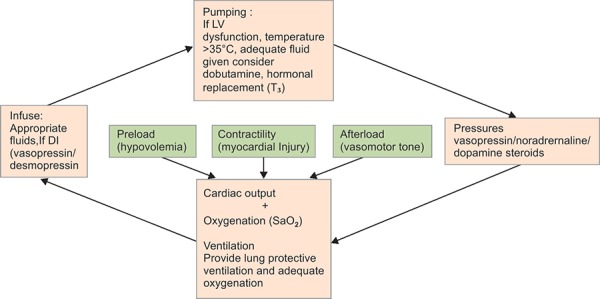
VIP approach for hemodynamic management of brain-dead patient

1. Volume expansion
2. Vasopressors and inotropes
3. Hormonal replacement

### Volume Expansion

#### Preferred Fluid for Resuscitation

The initial goal of fluid therapy in PBDD is intravascular volume replacement, so the fluid of choice is isotonic crystalloid. Initially 0.9% normal saline and ringers lactate are preferred.

But later on depending on hyperchloremic metabolic acidosis and hypernatremia fluid choice changes to half normal saline or 5% dextrose.^13^

Colliods mostly used as bolus infusions to tackle rapidly developing hypotension. Five percent albumin and hydroxylethyl starch are available (HES). HES is associated with acute kidney injury, coagulopathy and trapping of reticuloendothelial system. In delayed graft recipient functioning and graft failure was seen in renal transplant patients.^5,13^

Packed red cells should be transfused to achieve a hematocrit of 30% to maintain oxygen delivery.^5,13^

Liberal fluid balance/hydration with optimal mechanical ventilator settings are considered to be helpful for kidney procurement. But this might be hazardous for lung procurement.^5,13^

Techniques that can be used to assess effective fluid administration:^5,13^

- MAP at least 60 mm Hg
- Bedside echocardiography:
- Urine output 1–3 mL/kg/hour (rule out DI or diuretics)
- Decreasing dose of vasopressors

Other parameters like pulse pressure variation, cardiac index >2.5 (note – high cardiac output state due to vasodilatory shock may be a confounder) and central venous oxygen saturation >70% (note – low basal metabolism due to brain death may be a confounder) can be used to assess effectiveness of fluid administration.

### Vasopressor and Ionotropes

Vasoactive support is required secondary to brainstem death. It includes vasopressors, ionotropes and hormonal therapy.

Vasopressor may be required frequently to support mean arterial pressure once hypovolemia has been excluded/corrected.

#### Vasopressor of Choice

- Vasopressin in pressor dose (1–2 IU/hour) stabilizes the vasodialatory shock caused by brainstem death. Vasopressin up to 2.4 units/hour may reduce the requirement of other vasoactive agents. The use of low-dose vasopressin counteracts DI, aids restoration of vascular tone by stimulation of V1a receptors, and reduce epinephrine requirement dose. Hence selected as first or second line vasopressor in management of brain-dead patients.^5^
  Canadian guidelines recommend vasopressin as the first choice vasopressor for donor resuscitation.
- Norepinephrine is also commonly used for this purpose. It has potent α-receptor agonist activity as compared to dopamine. This predisposes to increased pulmonary capillary permeability, increasing extravascular lung water and coronary and mesenteric vasoconstriction and increase the afterload of the left heart.^5,13^
- *Dopamine*: Dopamine protects against ischemia/reperfusion injury and inflammation by induction of enzyme heme oxygenase-1. It was been observed that dopamine helps in faster alveolar fluid clearance and reduces the dialysis need in post kidney transplant recipient.^13^ But chances of arrthymias increase with higher dose of dopamine.^5^
- Dobutamine, epinephrine may be used in primary cardiac dysfunction.

### Hormonal Replacement Therapy (HRT) May Be Initiated If ^5,8,13^

- Hemodynamic goals are not met
- Left ventricular ejection fraction remains less than 45% (See section of HRT).

### Monitoring Tools

Monitoring tools are necessary in hemodynamic management. The monitoring devices required are to be tailor made as per the organ considered for procurement/ as per availability in the institute.

Monitoring tools are based on assessment of global tissue oxygen supply-demand markers. Thus, devices such as central venous catheter or pulmonary artery catheter and arterial line are recommended. This device provide hemodynamic parameters such as central venous pressure (CVP), pulmonary artery occlusion pressure (PAOP), MAP, cardiac output (CO) and cardiac index. Other devices can be used to calculate –CO, stroke volume (SV), stroke volume variation (SVV), cardiac index (CI) and mixed venous saturation may be valuable for guiding fluid management.

For fluid resuscitation arterial pulse pressure variation (PPV), stroke volume variation (SVV) variability of diameter of venacava, respiratory occlusion test, passive leg raising test and finally thoracic echocardiaography to calculate velocity- time integral in left ventricular outflow tract can be used. Each hospital have to develop protocol as per avialbility and as per patient requirement.

Most important bedside noninvasive device is transthroacic echocardiagraphy (TTE). This device will guide us for fluid resuscitation, fluid overload (lung sonography), cardiac contractility and ejection fraction.^5,13^

TTE will also help to assess condition of myocardium, valves, pericardium for cardiac transplantation consideration. Initial cardiac dysfunction may be due to brainstem death pathophysiology. The echocardiography need to be repeated (12–2 hours) following aggressive donor management or once vasopressor doses are reduced to minimum.^13^

When transthoracic echocardiography cannot be done due chest wall abnormalities or if cardiac functions need to be assessed more accurately then transoesophageal echocardiography (TEE) should be considered.

### Endocrine Dysfunction

The hypothalamus-pituitary axis gets affected in brainstem death patient. Reduction in vasopressin levels leads to diabetes insipidus(DI). Anterior pituitary hormones deficits results in hypothyroidism and hypocortisolism.

#### Diabetes Insipidus

It occurs in about 80% of the patients with brain death. However, absence of DI does not mean that the patient is not brain dead. DI results from the deficiency of antidiuretic hormone (arginine vasopressin-AVP) due to loss of posterior pituitary function. DI is associated with polyuria leading to hypovolemia, hyperosmolality and hypernatremia.^13^

Hypernatremia may adversely affect the outcomes for renal and liver transplants.^5,13,14^ Early use of antidiuretic agents in suspected DI may prevent physiological instability due to hypovolemia and hypothermia. Other causes of polyuria, such as hyperglycemia or the use of a diuretic or osmotherapy should be excluded before considering a diagnosis of DI.^5,13,14^

DI is likely to be present if one or more of the following criteria are identified in absence of other causes mentioned above^8,13,14^ (Table 4).

### Management of DI

- Fluid should be replaced by balanced salt solution to correct hypotension. If further correction of hypernatremia is required, once volume status is stabilized, hypotonic fluids with low-sodium content (5% dextrose or 0.45% saline) should be considered with a close watch for hyperglycemia.^5,13,14^

**Table 4:** Signs of DI

*Polyuria*: (>3 L/d or 2.5–3 mL/h/kg)
*Hyper natremia*: (>150 mEq/L)
*Low urinary specific gravity*: (<1.005)
*Urine hypo osmololarity*: (<200 mOsm/L)
*Serum hyper osmolarity*: (>295 mOsm/L)
Hypo kalemia, Hypo calcemia, Hypo magnesemia, Hypo phosphatemia
Dehydration

- Central diabetes insipidus (DI) should be treated with desmopressin or vasopressin depending on the patient's clinical status.
  – In donor presenting with hypotension. Start intravenous AVP at 0.01–0.04I U/minutes.^13^ *Or*
    Vasopressin IV infusion at a dose of 0.5–2.0 U/hour is indicated when CDI occurs in association with hypotension refractory to fluid resuscitation; it acts equally at all three vasopressin receptors, so has pressor effects in addition to antidiuretic actions. Aim is to maintain sodium level between 135–145 mEq/L but no more than 155 mEq/L during the management of CDI because some studies report worse liver graft survival with higher concentrations.^8^
  – For DI with hypernatremia without hypotension desmopressin (1-deamino-8-d-arginine vasopressin), a vasopressin analogue with greater affinity for the V2 receptor can be used.^5^

*Dosage*: An initial IV dose of desmopressin of 1–4 μg is used and subsequntly dose is titrated as per urine volume, serum sodium concentration and urine osmolality. Additional doses (1–2 µg every 6 hours) may be required.^5,13,14^

#### Thyroid Hormone Deficiency

Circulating tri-iodothyronine (T3) may be low in patients with brain death. Thyroid hormone deficiency along with cortisol deficiency may contribute to hemodynamic instability. Sometimes function of anterior pituitary is partially preserved, with normal levels of cortisol and thyroid hormone, or low thyroid hormone with normal/raised thyroidstimulating hormone levels consistent with sick euthyroid syndrome. Thus abnormal thyroid functions are seen after brain death is consistent with sick euthyriod syndrome rather than true hypothyroidism.

Recommendations for thyroid hormone replacement in brain death management is controversial as positive results have not been reported.^13^

Thyriodhormone replacement may only be needed for hemodynamically unstable donor and if cardiac function does not improve inspite of fluid resuscitation and vasopressor.

(See HRT section, for thyroid harmone replacement)

#### Corticosteriod Deficiency

The presumed reason for corticosteroid deficiency is firstly hypothalamic-pituitary-adrenal (HPA) axis failure which could mediate hemodynamic instability. Secondly hemodynamic instability and hormonal imbalances in brain-dead donor leads to the release of proinflammatory and immunological mediators. This has been associated with reduced graft function.

High doses of corticosteroids may reduce brainstem death-induced inflammation and help to modulate immune function. This improves the donor organ quality and post-transplant graft functioning. This has also shown to improve donor lung quality.^5,13,14^

#### Hyperglycemia

Brain death causes hormonal imbalances that result in reduced insulin synthesis and increased insulin resistance and gluconeogenesis. If untreated, hyperglycemia may cause pancreatic cell damage, which may affect pancreatic graft; therefore, measures should be taken to maintain strict euglycemia to minimize this risk.^14^

Insulin infusion should be initiated to control sugars. Blood sugars must be maintained in the range of 80–150 mg/dL.^14^

### Hormonal Replacement Therapy^5,13^

- *Vasopressin*: 1 U bolus followed by an infusion of 0.5–4.0 U/hour.^5^
- *Methylprednisolone*: 15 mg/kg immediately after the diagnosis of brain death and 24th hourly thereafter. Another option is 250 mg bolus, followed by 100 mg/hour inffusion till the organ retrieval.^5,8^
- **Insulin** infusion to maintain blood glucose between 80 and 150 mg.^5^
- *Thyriod replacement therapy*: It is replaced either alone or in combination hormone therapy with corticosteriod, vasopressin and insulin.

T4 20 mcg IV bolus followed by infusions of 10 mcg/hour. Or

T3 given as a 4.0 µg bolus followed by an infusion of 3 µ/hour. T4 improves hemodynamics and prevents cardiovascular collapse in hemodynamically unstable organ donors. However, intravenous T3 is generally not available. So, T4 oral 300–400 mcg/8 hourly is suggested instead of T3 (NOTTO).^5,6^

### Respiratory System

Neurogenic pulmonary edema (NPE) and inflammatory acute lung injury are the two main factors causing respiratory failure in brainstem death patients. Other contributing factors for lung dysfunction include chest trauma, aspirationatelectasis and nosocomial pnuemonia.^16^ This causes acute respiratory distress syndrome (ARDS) like picture. This could impede the potential for lung donation, and disturb the homeostasis of other organs.

### Management

Management of the potential donor is thus aimed at maintaining gas exchange to protect other organs, while taking care to preserve the lung. Lung protective ventilation/low stretch protocol: tidal volume 6–8 mL/kg of PBW, PEEP equal to 8–10 cm H_2_O, a closed circuit for tracheal suction, alveolar recruitment manoeuvres after any disconnection, and the use of continuous positive airway pressure during apnoea test.

Respiratory targets should be:^5,8^

- pH of 7.35–7.45
- Partial pressure of oxygen (PaO_2_) above 100 mm Hg with the use of the minimal fraction of inspired oxygen (FiO_2_)
- An oxygen saturation (SpO_2_) above 95%, and
- Partial pressure of carbon dioxide (PaCO_2_) of 35–40 mm Hg.

### Renal Changes

Renal tubular injuries are caused by proinflammatory and procoagulant effects secondary to brain death.^5,9,10,14^

*Management*: Appropriate fluid management and maintaining renal perfusion pressure. Fluid overload should be avoided. Oxygen saturation within normal limits and normocapnia should be maintained.^5,13^

### Hypothermia

Hypothermia occurs in brain death patients due to loss of thermoregulation, reduced metabolic rate, excessive heat loss, and loss of protective mechanisms such as vasoconstriction or shivering. Exposure and administration of cold fluids may further increase the risk of hypothermia.

Hypothermia has direct effect on cardiac function, arrhythmias, coagulation cascade, and oxygen delivery to tissues.

*Management of hypothermia*: Surface warming using warm blankets should be performed for all patients with hypothermia. Aim is to maintain a temperature over 35.8°C before and during the retrieval operation. Active warming can be achieved using warm blankets, fluid warmers, and heated humidifiers in ventilator circuits, administration of warm IV fluids and by adjusting the ambient temperature.^5,13,14^

### Anemia, Coagulopathy, and Immunological Changes^5^

Traumatic bleeding commonly results in anemia. Furthermore, coagulopathy and fluid administration may cause exacerbation of anemia. Significant rise in proinflammatory cytokines such as interleukin-6 has been observed in brain-dead organ donors which could be one of the causes of coagulopathy. It may be worsened due to hypothermia.

*Management*: It is recommended to maintain the hematocrit (Hct) above 30%. If the Hct drops below 30%, two units of packed red blood cells (PRBCs) should be transfused (ACC). Coagulopathy should be treated with blood products.

### Infection Management

Severe brain damage and cerebral death predisposes to infection, due to heavy impairment of the cellular immune system and hemodynamic instability, with consequent bacterial translocation from the bowel. Further the invasive lines used for monitoring purposes, mechanical ventilation, inadequate nursing, and medical management can increase chances of infection. Presence of infection may complicate the donor organs and further the recipient. The actual rate of unexpected infection transmission from donor to receptor is low, occurring in less than 1–5%^8,13,15^ of solid organ transplant recipients.

Bacteremia or sepsis are not contraindications to donation, provided pathogen specific antibiotics have been administered for at least 48 hours prior to procurement.^13,15^ The infection screening protocols should be implemented and they vary as per geographical regions and transplant centers.^16^

#### Donor with Bacteremia and/or Sepsis/or with Positive Blood Cultures: Is It a Contraindication?

Positive blood cultures in potential organ donor is not unusual and the documented rate is 20% in literature^13,15,16,^ The incidence of positive blood culture appears to be more in older donors (>50 years) and in donors whose length of stay is more than 3 days.^13^ In younger donors (<40 years) bacteremia is most likely due to gram positive microbes (*Staphylococcus aureus*), while in olders it is due to gram negative bacteremia.^13^

**Table 5:** General care for brain-dead patients

Hand hygiene Central line and arterial line insertion and monitoring Nasogastric tube insertion Foley's catheter insertion Care of lines, Foley's catheter Propped up position - 30°–40° elevation Frequent change in body positioning (every 2 hours) Warming blankets to maintain body temperature around 36.5°C Prophylaxis for deep vein thrombosis - pneumatic compression device Eye care – tapping of eyelids Tracheal toileting: frequent airway suctioning Stress ulcer prophylaxis Broad spectrum antibiotics (to be prescribed as per hospital antibiotic stewardship programme)

But rate of transmission of infection to recipient is less,^18^ provided donor organ procurement is delayed for 48 hours so that donor receives antibiotic as per culture sensitivity for 48 hours.^13,18^

#### If the Blood Cultures of Donor Comes Positive after the Organ Transplantation

This should be communicated with the transplant intensivist/ transplant surgeon, so that the recipient receives pathogen specific antibiotics for 7–10 days.^19^ Ruiz et al. have reported equivalent graft and survival outcomes in these recipient by using standard post transplant antibiotic regime.^20^

#### Can PBDD with Meningitis Donate Organs?

Potential organ donor with bacterial meningitis are considered suitable for organ procurement only if they receive pathogen specific antibiotic or therapy against the probable pathogen. The suggested duration of therapy is same as above (48 hours) and the recipient should receive similar antibiotics for 5–10 days.^21^

PBDD with undiagnosed febrile illness, encephalitis, meningitis or flaccid paralysis of unknown cause and lymphocytic choriomeningitis virus and rabies are absolute contraindication for organ donation.

### General Care

*Summary*: Suggested protocolized ABC approach for management of potential brainstem death patient in Indian perspective (Table 5).^5,10,13^

### ABC Care for Organ Donor

A: ETIOLOGY of brainstem death or irreversible coma; B: BRAIN death alert signs; C: CONTRAINDICATION to organ donation

## CONCLUSION

With increasing mismatch between organs availability and need, all hospitals involved in cadaver organ donation programs have to keep necessary policies and protocols ready for effective implementation and to promote organ donation program. Clinical checklists should be followed for giving care to brian dead victims. In order to increase the rate of organ donation, multi retrieval of various organs and to maintain the quality of donated organs, we strongly suggest that periodic training programs should be in place for healthcare workers involved in organ donation process.
